# Economic Value of Peer Support Program in German Hospitals

**DOI:** 10.3389/ijph.2024.1607218

**Published:** 2024-06-13

**Authors:** Hannah Roesner, Thomas Neusius, Reinhard Strametz, José Joaquín Mira

**Affiliations:** ^1^ Hochschule RheinMain, Wiesbaden, Germany; ^2^ Miguel Hernández University of Elche, Elche, Spain; ^3^ Department of Health Psychology, Miguel Hernández University, Elche, Spain

**Keywords:** patient safety, peer support program, second victim, health worker safety, economic impact

## Abstract

**Objectives:**

Acknowledging peer support as the cornerstone in mitigating the psychosocial burden arising from the second victim phenomenon, this study assesses the economic benefits of a Peer Support Program (PSP), compared to data of the Resilience In Stressful Events (RISE) program in the US, within the acute inpatient care sector in Germany.

**Methods:**

Employing a Markov model, this economic evaluation analyzes the cost benefits, including sick day and dropout costs, over a 1-year period, comparing scenarios with and without the Peer Support Program from a hospital perspective. The costs were calculated as an example based on a hospital with 1,000 employees. The estimations are considered conservative.

**Results:**

The anticipated outcomes demonstrate an average cost saving of €6,672 per healthcare worker participating in the Peer Support Program, leading to an annual budgetary impact of approximately €6,67 Mio. for the studied hospital.

**Conclusion:**

The integration of a PSP proves economically advantageous for German hospitals, not only preserving financial resources but also reducing absenteeism, and mitigating turnover, thereby enhancing overall patient care.

## Introduction

The healthcare profession inevitably exposes practitioners to highly stressful events. Healthcare providers involved in unanticipated adverse patient events, unintentional healthcare errors, or patient injuries, and who become negatively impacted, are defined as “second victims” [1]. Prevalence studies among German nurses and physicians revealed a 59%–60% prevalence of second victims, with a 12-month prevalence of 49% for nurses and 35% for physicians [2, 3]. Emotional reactions, coping strategies, and overall wellbeing post-event vary widely among individuals [4–6]. The resulting spectrum of psychological responses includes guilt, anxiety, diminished self-confidence, loss of trust in the healthcare system, absenteeism, turnover intentions, alcoholism, and, in extreme cases, suicide [7–9].

The second victim phenomenon not only negatively affects individuals but also has the potential to detrimentally impact the quality of future patient care [10]. This impact may manifest through defensive medical practices or an elevated incidence of medical errors following post-traumatic stress disorder development [11–14]. The recovery process may lead second victims down paths of dropping out, surviving, or thriving [15], with outcomes affecting work positivity, time off, or even departure from the profession [15, 16]. These outcomes not only harm individuals but also result in financial losses for employing institutions. Nurse turnover, a significant challenge for the healthcare sector, leads to intellectual capital and productivity losses [17–19]. Supportive interventions can alleviate the negative consequences of the second victim phenomenon [20]. However, a lack of institutional support is reported by the majority of healthcare providers [15, 21]. The need for structured support programs is evident from surveys in Europe [22, 23] and the US [13, 15, 24, 25].

While other countries have established support program examples, such as RISE [21], the forYOU program [26] and the Medically Induced Trauma Support Service (MITSS) program [27] in the US, the open access online Second Victim support program MISE (Mitigating the Impact on Second Victims) [28] in Spain, Kollegiale Hilfe (KoHi) in an Austrian hospital [29], and a support program in Switzerland [30], Germany has only initial voluntary commitment-based approaches.

The Joint Commission as an independent, non-profit organization that accredits US health programs and organizations [31], recommends healthcare institutions establish structured peer support programs (PSP), emphasizing proactive peer support [32]. Healthcare workers seem to mostly rely on persons they are close with, and to a much lesser extend seek professional help [33]. Peer support is identified as the most desired form of support by second victims [2, 7, 25, 34–36], with evaluations of program effectiveness in various studies [34, 35, 37].

In addition to positive medical and psychological effects, support programs for second victims in Germany are anticipated to be cost-effective. Moran et al.'s study on the Resilience in Stressful Events (RISE) program revealed potential savings of $1.81 million within a healthcare institution when applied to a staff of 80 nurses [38]. The RISE program, designed to help hospital staff cope with stressful patient-related events [21], demonstrated cost benefits by comparing program costs to reduced financial losses due to healthcare worker absenteeism. However, the economic impact of a PSP in Germany remains unexplored. To address this gap, we investigated the economic cost benefits of implementing a PSP in the acute inpatient care sector in Germany.

## Methods

### Design

To assess the economic cost benefits of a support program with consideration of macroeconomic effects, we employed health economic model calculations. This evaluation focused on support programs within the acute care nursing sector in Germany, specifically targeting an institution with 1,000 nursing staff, equivalent to a hospital with approximately 550–600 beds. Model parameters were derived from survey data from previous studies and expert judgement, if empirical evidence was unavailable or unconvincing. A Markov chain model based on single day cycles was developed, allowing to determine expectation values on a time horizon of 1 year, i.e. 365 daily cycles. Stochastic modeling allowed us to assess the model’s sensitivity to parameter variations. Costs were reported in Euros, and the time horizon for the analysis was 1 year, concentrating on nursing staff for comparability with other studies. Direct costs such as the time off and worker replacement costs in acute inpatient care sector in Germany are considered, whereas indirect costs like employer productivity losses or quality impairments in the work of affected staff members were not considered.

### Model

**FIGURE 1:**
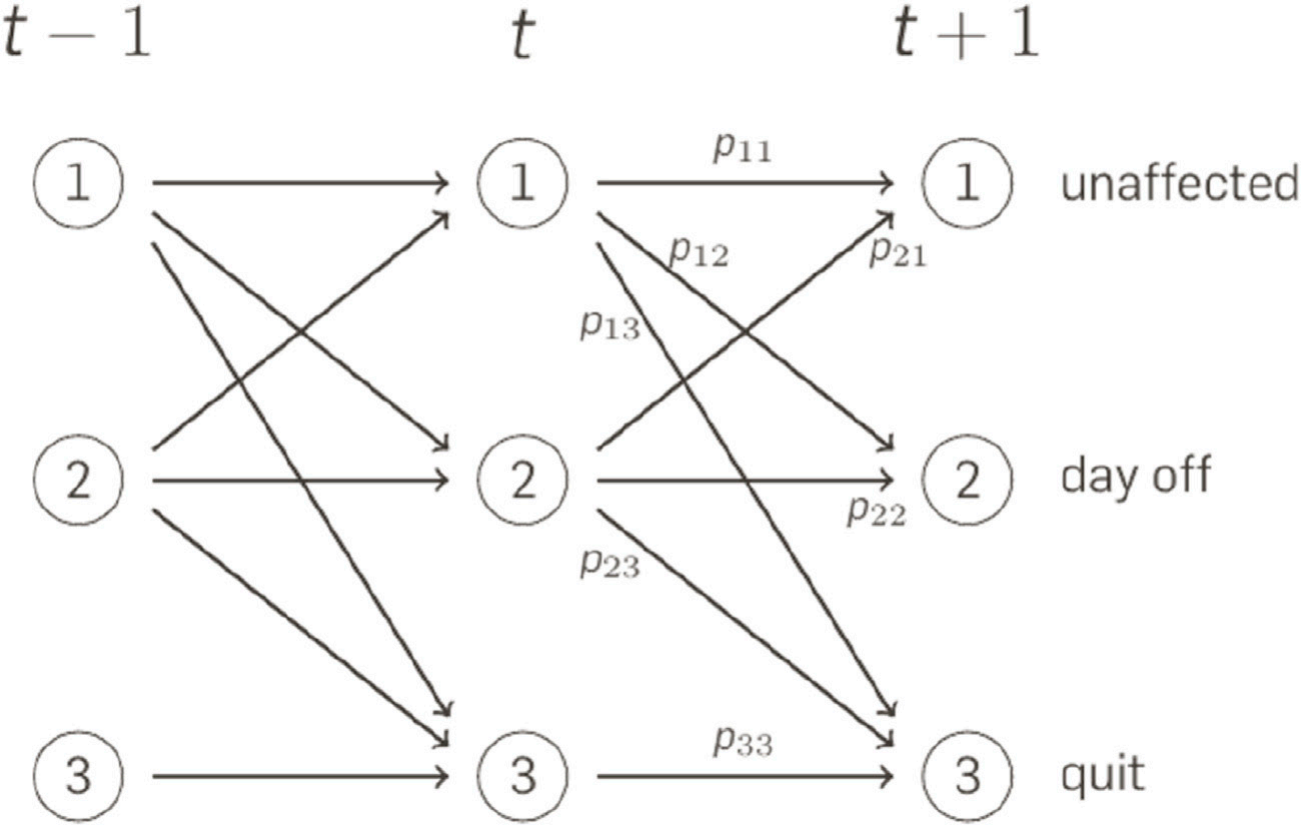
Markov Chain Model: At every time step t, the individual is either [1] unaffected [2], takes a day off, or [3] quits the job. The pij define the transition probability from state i to state j in the subsequent time step. Economic Value of Peer Support Program in German Hospitals, Germany, 2024.

Building upon Moran et al.’s study [38], we constructed a Markov chain model (Figure 1). Markov chains describe a time series of events in discrete steps. The probability to reach a given state at time *t* depends only on the state in the previous time step *t-1*. To reach state *j*, after being in state *i* in the previous step, is referred to as *p*_*ij.*_ The model describes the state of an individual as being one of three possibilities, that are (a) unaffected, (b) 1 day leave, (c) quit, the latter two of which being identified with financial losses. The transition between these states from day to day were conditioned on the random event of a stressful incident (high impact event, HIE). Every individual had each day an identical risk of being exposed to a HIE independently of previous occurrences. The model operated on a daily cycle, spanning 365 cycles in total. If nurses chose to quit, they permanently exited the modeling cycle. In contrast to Moran et al., we include the duration of HIE induced leaves by assuming that individuals return to the unaffected state with a reduced probability. The Markov chain allows a deterministic description of the expectation value of losses.

### Assumptions

Our calculations assume that each employee faces an unforeseen incident daily, i.e., HIE, with a probability of 2.00%. Upon an HIE, the probability of a sick leave increases to 5.00% (compared to 0.03% without trauma). Likewise, the probability of resignation rises to 0.68% (compared to 0.03% without trauma). The introduction of a PSP program reduces the probability of a sick leave to 3.00% and the probability of resignation to 0.34%. The assumptions are based on Moran et al. (2020), but adapted to the present situation (Tables 1, 2). In particular, we assume a far lower incidence rate and a lower probability to quit, but a higher probability of sick leaves with an average length of 7 days (corresponding to a recovery rate of 14.29% per day).

**TABLE 1:** Probabilities of employees upon a critical event. Economic Value of Peer Support Program in German Hospitals, Germany, 2024.

State	Base case
Probabilities High-impact event	0.0200
No PSP	
- Day off (high impact)	0.0500
- Day off (low impact)	0.0020
- Quit (high impact)	0.0068
- Quit (low impact)	0.0003
PSP	
- Day off (high impact)	0.0300
- Day off (low impact)	0.0020
- Quit (high impact)	0.0034
- Quit (low impact)	0.0001

**TABLE 2:** Transition probabilities greater zero, as obtained from combining the high impact event (HIE) incidence rate with the probabilities of taking a day off/quitting. Economic Value of Peer Support Program in German Hospitals, Germany, 2024.

	No PSP	With PSP
P_11_	0.9966	0.9973
P_12_	0.0030	0.0026
P_13_	0.0004	0.0002
P_21_	0.1429	0.1429
P_22_	0.8571	0.8571
P_33_	1.0000	1.0000

According to expert judgement, the loss of a 1-day leave is assumed to be €500 and the replacement of a nurse that quits accounts to €75,000. The estimated cost associated with participating in the support program is €550 per healthcare worker within 1 year.

The model ignores HIE effects on productivity of impaired staff members.

### Sensitivity Analysis

To assess sensitivities, we run the model for 100,000 pairs of individual trajectories, each pair consisting of a PSP and non PSP variant, varying transition probabilities and expenditures for each pair such that the parameters were normally distributed around the base case values of the model with a standard deviation of 10% of the distribution’s expectation value and a lower bound of zero.

## Results

The simulation encompassed direct costs per sick day for healthcare workers and recruitment costs for new employees approximated by annual salaries. In the course of 1 year 14.3% of the nursing staff quit the job, whereas the introduction of PSP reduced this figure to 5.8%. Sick days are moderately reduced from an expected value of 6.77 days without PSP to 6.14 days with PSP.

The economic model calculation for a healthcare facility in Germany, mirroring the RISE program at Johns Hopkins University, is presented in Table 3. These figures were calculated in consideration of the above event probabilities for 1,000 individuals. Introducing a support program in the simulation resulted in an increase in both the number and cost of sick days, attributable in part to the significantly reduced number of dropouts, leading to more employees remaining with the organization.

**TABLE 3:** Effects in an Institution with 1,000 employees upon implementing a Peer Support Program (PSP). Economic Value of Peer Support Program in German Hospitals, Germany, 2024.

Information per year	Without PSP	With PSP
Sick days	6766	6141
Dropouts	143	58
Cost of sick days	3,383,230 €	3,070,470 €
Cost of dropouts	10,694,785 €	4,335,666 €
Total costs	14,078,015 €	7,406,136 €
Cost per Person	14,078 €	7,406 €

Considering the costs associated with participating in a PSP (approximately €550), the avoidance of sick days in specific cases (€500/day), and the costs of refilling a position in case of dropout (€75,000), an average cost saving of €6,672 per healthcare worker participating in the support program was determined using a three-stage Markov model, compared to non-participation. Main reason for the reduction is the reduction of dropouts, whereas the costs of sick day leaves are only moderately affected. The expected annual budgetary impact of implementing the support program is estimated to be approximately €6,67 Mio in the considered hospital. Additionally, the anticipated benefits of the support program, apart from reduced absenteeism, stem from increased job satisfaction, and lower staff turnover, ultimately enhancing patient care and preserving the hospital’s financial resources.

### Sensitivity Analysis

A number 100,000 stochastic trajectories were generated of pairs (non-PSP, PSP) scenarios. 29.0% of the non-PSP trajectories remained without any HIE-related effects (no sick leaves, no dropout). The figure rose to 36.9% in the PSP case. The 95% quantile of HIE-related costs was €79,443 without PSP, whereas the PSP scenarios exhibit 95% quantile of €68,222.

To test the robustness of the result in light of the uncertainties of our model parameters, we performed a Wilcoxon sign-ranked test. We applied the test in the one-sided version, with the null hypothesis that costs are higher without PSP than with PSP implemented. The hypothesis is significantly violated (*p* < 0.0001). Even after shifting the costs of the PSP variant homogeneously by adding an additional amount of 1,358.50 EUR, the hypothesis still can be rejected significantly (*p* = 0.0490).

## Discussion

Healthcare professionals need to be supported in order to be able to provide quality care after a HIE. Beyond positive medical and psychological outcomes, the provision of support services in hospitals has the potential for cost-effectiveness, a facet not previously evaluated in Germany. This study represents the first investigation, to our knowledge, into the economic impact of a PSP in a European hospital. By adapting a Markov Chain Model for the implementation of a PSP in the acute inpatient care sector in Germany, our results demonstrate substantial cost savings for the hospital, constituting significant value.

We estimated that the existence of a PSP in a hospital with 1,000 nursing employees in Germany enables savings of €6,67 Mio annually. These findings align with a cost-analysis conducted at Johns Hopkins Hospital, revealing potential savings of $1.81 million from the RISE program in a smaller sample of 80 nurses [38]. The main driver of the above result is the reduction of the probability to quit the job. Our assumption of a reduction from 0.68% to 0.34% is considerably smaller than the one in Moran et al., who assumed a drop from 1.22% to 0.34%. Therefore, we consider our results to be conservative.

The expected budgetary impact within the institution indicates economic potential even in medium-sized companies with 1,000 employees, despite not considering subsequent costs arising from reduced performance and indirect costs associated with unsafe work, estimated at 13% of total healthcare expenditure according to the OECD’s recent publication in “The Economics of Patient Safety” [39], in our approximate model.

The calculations of the Markov Model resulted in the assumption that 14.3% of nursing staff quit their job in the course of a year. According to the German Hospital Report 2021 [40], this is in line with the current figures for staff turnover in German hospitals, where one in six change jobs every year [41]. The implementation of a PSP within the simulation exhibited a reduction of dropouts by 8.5% and a notable rise in both the quantity and financial impact of sick days. This increase of sick days can be attributed partially to a marked reduction in the number of dropouts, consequently fostering greater retention of employees within the organization.

The implementation of psychosocial programs for healthcare workers in hospitals has been shown to have a positive impact on employee wellbeing and organizational outcomes [42]. The role of employee programs and psychological wellbeing are important factors influencing job satisfaction, ultimately contributing to employee retention. PSPs are likely to facilitate hospitals in reducing turnover rates, improve healthcare worker resilience and enhancing the quality of care [43–45]. For instance, the implementation of such programs could increase the probability of healthcare providers arriving at work in an optimal state of wellbeing, thereby fostering a positive work environment conducive to the delivery of high-quality, safe care. Furthermore, providers may exhibit greater engagement and a heightened commitment to the organization as a result.

Our findings align with prior studies suggesting that hospitals with peer support positively impact employee retention [46, 47] and hospitals with poor nurse retention spend more than those with high retention [48]. While improving employee wellbeing contributes to reducing healthcare expenditure by minimizing the cost of work-related harm by up to an estimated 2% of healthcare expenditure, it also contributes to minimizing patient harm by up to an estimated 12% [49].

The occurrence of events leading to second victims can have cascading effects, including burnout and elevated turnover rates among healthcare providers, ultimately exerting adverse influences on the quality of future patient care and financial impacts on the hospital. However, comprehensive and easily accessible support programs tailored to healthcare providers in Germany remain absent on a national scale. Our research contributes to the existing evidence endorsing the integration of institutional PSP for healthcare providers into hospitals. We demonstrate that such adoption may yield financial advantages for hospitals, thereby further strengthening the case for their implementation.

The absolute values presented in this study are specific to Germany but the model can be universally applied once relevant data on sickness absence rates and personnel replacement costs are available. This underscores the adaptability and versatility of the model in assessing the economic impact of PSP on hospitals worldwide. By accounting for local variations in wage structures and wage replacement modalities, policymakers, managers, and businesses can utilize this model to adapt and tailor interventions and strategies aimed at mitigating the adverse effects of second victim phenomenon in their respective regions.

The implementation of a PSP presents a proactive approach in addressing the inevitability of medical errors within clinical practice. Through widespread adoption across German hospitals, healthcare professionals gain immediate access to support following HIE, enabling them to recover and maintain quality patient care delivery. While the introduction of such programs incurs costs, the strategic provision of targeted support services aids affected individuals in managing negative consequences, ultimately yielding long-term economic benefits for the institution. The findings of this research indicate that implementing a PSP for medical providers could yield a significant return on investment for hospitals, thereby representing a beneficial value proposition for the healthcare institution.

### Limitations

This study has limitations. Examining the precise impact of adverse events on healthcare professionals’ decision to leave their medical or nursing roles is challenging due to the multifaceted nature of underlying factors. Whilst this study is focused on second victims, the impacts and consequences on their working environment are not considered. Reliable isolation of the effects of adverse events necessitates large-scale and resource-intensive studies, which might be difficult to conduct. The scope of this study shows a simplified perspective due to the scarcity of comprehensive data. Our analysis draws from available data, existing literature, and expert opinions. Indirect costs are not taken into account in the calculation and our expectations concerning the positive impact of PSP are cautious, which makes our results a conservative estimate and thus potentially underestimates the final outcome. Also not accounted for in the calculation were other professional groups that would also benefit from a support program. Findings and conclusions should be interpreted with caution, as the context and effects of peer support in healthcare settings may vary considerably.

### Conclusion

Using a three-stage Markov model, our study reveals an average cost saving of €6,672 per healthcare worker participating in the support program compared to non-participation. While the absolute values may vary, the underlying framework remains universally applicable, enabling cross-country comparisons and informed decision-making in assessing the economic effects of support programs and addressing healthcare worker safety related challenges. Hospital managers are encouraged to recognize the advantages of cost savings and reduced staff turnover associated with establishing PSP, thereby addressing the critical issue of the second victim phenomenon in healthcare and improving health worker and patient safety. Systematic support, particularly by healthcare organizations and institutions, is crucial. Therefore, further studies on effective and immediate supervision and support strategies, along with legal frameworks in Germany, are needed to mitigate the adverse effects of unforeseen incidents on a Second Victim.
